# Supplementary material for: State-of-the-Art Quantum Chemistry Meets Variable Reaction Coordinate Transition State Theory to Solve the Puzzling Case of the H2S + Cl System
Source: J Chem Theory Comput. 2020 Jun 30;16(8):5090–104. doi: 10.1021/acs.jctc.0c00354 (PMC8009477; doi:10.1021/acs.jctc.0c00354)
Supplement: Supplementary file 1 — ct0c00354_si_001.pdf [file ct0c00354_si_001.pdf]

Suppporting Information

State-of-the-art quantum chemistry meets variable reaction coordinate transition state theory to solve the puzzling case of the $H_2S + Cl$ system

Jacopo Lupi,† Cristina Puzzarini,‡ Carlo Cavallotti,¶ and Vincenzo Barone*,†

†*Scuola Normale Superiore, Piazza dei Cavalieri 7, I-56126 Pisa, Italy*

‡*Department of Chemistry "Giacomo Ciamician", University of Bologna, Via F. Selmi 2, I-40126 Bologna, Italy*

¶*Department of Chemistry, Materials, and Chemical Engineering "G. Natta", Politecnico di Milano, I-20131 Milano, Italy*

E-mail: vincenzo.barone@sns.it

Input file for RRKM-ME calculations using the MESS package for the $H_2S + Cl$ reaction

In the following, the input file for the VRC-VTSTin calculation is reported. All the important molecular properties are enclosed. The nomenclature used is the same used in Section 3.4.

VRC-VTSTin

```
!***************************************************
!               GLOBAL SECTION
!***************************************************
!!!!!!!!!!!!!!!!!!!!!!!!!!!!!!!!!!!!!!!!!!!!!!!!!!!!
!
!
TemperatureList[K]                      200 225 250 275 300 325 350 375 400   425 430 450 475 500 600 700 800 900 1000
PressureList[atm]                       0.5 1
!
!
EnergyStepOverTemperature               .2         ! [Discretization energy step (global relax matrix)] / T
ExcessEnergyOverTemperature             30         ! [Highest barrier in the model (global relax matrix)] / T
ModelEnergyLimit[kcal/mol]              400        ! Highest reference energy used in the calculation ( or ReferenceEnergy[kcal/mol])
!
CalculationMethod                       direct     ! direct or low-eigenvalue
!
WellCutoff                              20         ! well truncation parameter : Max { dissociation limit (min barrier rel. to bottom of the well) / T }
ChemicalEigenvalueMax                   0.2        ! Max chemical eigenvalue / Lowest Collision relaxation eigenvalue
!
ReductionMethod                         diagonalization ! [low eigenvalue method only] diagonalization or projection (default)
!
!!!!!!!!!test!!!!!!!!!!!!!!!!!!!
!WellCutoff                             10
!ChemicalEigenvalueMin                  1.e-6          #only for direct diagonalization method
!!!!!!!!test!!!!!!!!!!!!!!!!!!!!!!!!
AtomDistanceMin[bohr]                   1.3
!!
RateOutput                              rate.out                     ! output file name for rate coefficients
!
!
!!!!!!!!!!!!!!!!!!!!!!!!!!!!!!!!!!!!!!!!!!!!!!!!!!!!
!***************************************************
!               MODEL SECTION
!***************************************************
!!!!!!!!!!!!!!!!!!!!!!!!!!!!!!!!!!!!!!!!!!!!!!!!!!!!
!
!
Model
!
  EnergyRelaxation                                                   ! Default collisional energy relaxation kernel
    Exponential                                                      ! Currently the only possible energy relaxation model
       Factor[1/cm]                     260                          ! (Delta_E_down)^(0) @ standard T (300 K)
       Power                            0.875                        ! Power n in the expression (Delta_E_down) = (Delta_E_down)^(0) (T/T0)^(n)
       ExponentCutoff                   10                           ! if (Delta_E) / (Delta_E_down) > value  transition probability is zero
    End
!
  CollisionFrequency                                                 ! Collision frequency model
    LennardJones                                                     ! Currently the only possible collisional frequency model  based on LJ potential
       Epsilons[K]                       90.58  617.0                ! Epsilon_1 and Epsilon_2
       Sigmas[angstrom]                  3.54    5.62                ! Sigma_1 and Sigma_2
       Masses[amu]                       39.948 69.0                 ! Masses of the buffer gas molecule and of the complex (check order)
   End
!
!*************************************************
```

```
!
!****************************************************
!!!!!!!!!!!!!!!!!!!!!!!!!!!!!!!!!!!!!!!!!!!!!!!!!!!!!
!****************************************************
!  REACTANTS
!****************************************************


 Bimolecular REACS
 Fragment REACT1
 RRHO
 Geometry[angstrom]              3
   S                 0.00000000    0.00000000    0.10283700
   H                 0.00000000    0.96718500   -0.82269200
   H                 0.00000000   -0.96718500   -0.82269200
  Core  RigidRotor
  SymmetryFactor    2.0000000000000000
  End
     Frequencies[1/cm]           3
   1217.89 2735.01 2750.20
  ZeroEnergy[kcal/mol]            0.
  ElectronicLevels[1/cm]                     1
   0           1
 !************************************
 End
!*********
 Fragment REACT2
  Atom
  Name Cl
  ElectronicLevels[1/cm]                     2
   0       4
   881.00  2

 !************************************
 End
!*********
GroundEnergy[kcal/mol] 0.0
End
 Bimolecular PRODS
 Fragment PROD1
 RRHO
 Geometry[angstrom]              2
   H                 0.00000000    0.00000000   -1.26323400
   S                 0.00000000    0.00000000    0.07895200
  Core  RigidRotor
  SymmetryFactor    1.0000000000000000
  End
     Frequencies[1/cm]           1
   2719.96
  ZeroEnergy[kcal/mol]            0.
  ElectronicLevels[1/cm]                     2
   0.0000000000000000        2.0000000000000000
   376.95999999999998        2.0000000000000000
 !************************************
 End
!*********
```

```
 Fragment PROD2
 RRHO
 Geometry[angstrom]               2
   H                  0.00000000    0.00000000   -1.20561900
   Cl                 0.00000000    0.00000000    0.07091900
  Core  RigidRotor
  SymmetryFactor    1.0000000000000000
  End
     Frequencies[1/cm]            1
   3006.51
  ZeroEnergy[kcal/mol]             0.
  ElectronicLevels[1/cm]                       1
   0.0000000000000000        1.0000000000000000

 !************************************
 End
GroundEnergy[kcal/mol]   -12.00
End
!*********
 Well WR
 Species
 RRHO     ! well
 Geometry[angstrom]               4
   H                  0.86853200   -1.21794200    0.97069800
   S                 -0.05263800   -1.26295000    0.00000000
   H                  0.86853200   -1.21794200   -0.97069800
   Cl                -0.05263800    1.33194700    0.00000000
  Core  RigidRotor
  SymmetryFactor    1.0000000000000000
  End
     Frequencies[1/cm]            6
   235.50 361.30 394.60 1211.08 2735.16 2749.92
  ZeroEnergy[kcal/mol]     -8.58
  ElectronicLevels[1/cm]                       1
   0.0000000000000000        2.0000000000000000
 End
 End
 !************************************
 Well WP
 Species
 RRHO     ! well
 Geometry[angstrom]               4
    S                 -1.89837200   -0.07864900   -0.00000300
    H                 -1.96120000    1.26293700   -0.00000900
    H                  0.58255000   -0.07505800    0.00001500
    Cl                 1.86780000    0.00414800    0.00000200
   Core  RigidRotor
   SymmetryFactor    1.0000000000000000
  End
    Frequencies[1/cm]            6
   92.55 144.45 254.96 397.14 2715.09 2844.56
  ZeroEnergy[kcal/mol]              -13.88
  ElectronicLevels[1/cm]                       1
   0.0000000000000000        2.0000000000000000
 End
 End
```

```
!************************************
Barrier B1  REACS WR
RRHO
   Stoichiometry H2S1Cl1
    Core Rotd
      File flux_lr_so9.dat
      SymmetryFactor    2.2200000000
    End
    Frequencies[1/cm]            3
  1217.89 2735.01 2750.20
   ZeroEnergy[kcal/mol]    0.0
    ElectronicLevels[1/cm]              1
  0.0000000000000000        2.0000000000000000
End
!************************************
Barrier B3  WP PRODS
  RRHO
   Stoichiometry  H2S1Cl1
   Core PhaseSpaceTheory
     FragmentGeometry[angstrom]              2
  H                  0.00000000    0.00000000   -1.26323400
  S                  0.00000000    0.00000000    0.07895200
     FragmentGeometry[angstrom]              2
  H                  0.00000000    0.00000000   -1.20561900
  Cl                 0.00000000    0.00000000    0.07091900
 SymmetryFactor     1.0000000000000000
         PotentialPrefactor[au]  230.
         PotentialPowerExponent  6
     End
   Frequencies[1/cm]           2
   2719.96 3006.51
ZeroEnergy[kcal/mol]    -12.00
 ElectronicLevels[1/cm]                  2
  0.0000000000000000        2.0000000000000000
  376.95999999999998        2.0000000000000000
End
!************************************
Barrier B2 WR WP
Variational
RRHO            !           90
Geometry[angstrom]           4
Cl   1.36787   0.01974  -0.00798
H   -0.27379  -0.19797   0.71945
S   -1.44332  -0.05571  -0.01648
H   -1.39984   1.28048   0.08033
 Core  RigidRotor
          SymmetryFactor   0.50000000000000000
End
    Frequencies[1/cm]            5
  2733.8      2093.0      1136.8      394.25      189.40
ZeroEnergy[kcal/mol]   -1.3891033240988111
 ElectronicLevels[1/cm]                     1
  0.0000000000000000        2.0000000000000000
End
!*********************************************
RRHO            !           91
```

```
Geometry[angstrom]           4
Cl   1.36762   0.01969  -0.00777
H   -0.26174  -0.19606   0.70931
S   -1.44342  -0.05572  -0.01638
H   -1.39997   1.28056   0.07988
 Core  RigidRotor
          SymmetryFactor   0.50000000000000000
End
    Frequencies[1/cm]           5
  2733.4      2039.3      1129.8      396.99      191.83
ZeroEnergy[kcal/mol]   -1.3246034340405763
 ElectronicLevels[1/cm]                     1
  0.0000000000000000        2.0000000000000000
End
!**********************************************
RRHO           !           92
Geometry[angstrom]           4
Cl   1.36736   0.01964  -0.00756
H   -0.24947  -0.19415   0.69944
S   -1.44353  -0.05573  -0.01629
H   -1.40008   1.28065   0.07946
 Core  RigidRotor
          SymmetryFactor   0.50000000000000000
End
    Frequencies[1/cm]           5
  2733.0      1982.1      1122.1      399.78      194.59
ZeroEnergy[kcal/mol]   -1.2720111512322105
 ElectronicLevels[1/cm]                     1
  0.0000000000000000        2.0000000000000000
End
!**********************************************
RRHO           !           93
Geometry[angstrom]           4
Cl   1.36710   0.01959  -0.00735
H   -0.23696  -0.19225   0.68988
S   -1.44364  -0.05574  -0.01620
H   -1.40016   1.28073   0.07908
 Core  RigidRotor
          SymmetryFactor   0.50000000000000000
End
    Frequencies[1/cm]           5
  2732.5      1921.6      1113.6      402.59      197.68
ZeroEnergy[kcal/mol]   -1.2310834484781283
 ElectronicLevels[1/cm]                     1
  0.0000000000000000        2.0000000000000000
End
!**********************************************
RRHO           !           94
Geometry[angstrom]           4
Cl   1.36684   0.01954  -0.00714
H   -0.22418  -0.19036   0.68068
S   -1.44375  -0.05574  -0.01612
H   -1.40021   1.28081   0.07873
 Core  RigidRotor
          SymmetryFactor   0.50000000000000000
End
```

```
    Frequencies[1/cm]            5
  2732.1      1858.4      1104.3      405.40      201.13
ZeroEnergy[kcal/mol]   -1.2010483570396526
 ElectronicLevels[1/cm]                      1
  0.0000000000000000        2.0000000000000000
End
!*********************************************
RRHO           !           95
Geometry[angstrom]           4
Cl   1.36658   0.01949  -0.00694
H   -0.21112  -0.18850   0.67187
S   -1.44388  -0.05575  -0.01606
H   -1.40024   1.28089   0.07841
 Core  RigidRotor
          SymmetryFactor   0.50000000000000000
End
    Frequencies[1/cm]            5
  2731.6      1793.2      1094.0      408.20      204.92
ZeroEnergy[kcal/mol]   -1.1871230884349664
 ElectronicLevels[1/cm]                      1
  0.0000000000000000        2.0000000000000000
End
!*********************************************
RRHO           !           96
Geometry[angstrom]           4
Cl   1.36633   0.01944  -0.00674
H   -0.19777  -0.18666   0.66349
S   -1.44402  -0.05576  -0.01600
H   -1.40024   1.28097   0.07812
 Core  RigidRotor
          SymmetryFactor   0.50000000000000000
End
    Frequencies[1/cm]            5
  2731.1      1727.3      1082.9      410.98      209.05
ZeroEnergy[kcal/mol]   -1.1814743148651377
 ElectronicLevels[1/cm]                      1
  0.0000000000000000        2.0000000000000000
End
!*********************************************
RRHO           !           97
Geometry[angstrom]           4
Cl   1.36608   0.01939  -0.00655
H   -0.18414  -0.18488   0.65558
S   -1.44418  -0.05577  -0.01596
H   -1.40022   1.28105   0.07786
 Core  RigidRotor
          SymmetryFactor   0.50000000000000000
End
    Frequencies[1/cm]            5
  2730.6      1661.8      1070.8      413.69      213.48
ZeroEnergy[kcal/mol]   -1.1950082180404342
 ElectronicLevels[1/cm]                      1
  0.0000000000000000        2.0000000000000000
End
!*********************************************
RRHO           !           98
```

```
Geometry[angstrom]           4
Cl   1.36583   0.01935  -0.00636
H   -0.17022  -0.18315   0.64815
S   -1.44435  -0.05577  -0.01593
H   -1.40017   1.28112   0.07763
 Core  RigidRotor
          SymmetryFactor   0.50000000000000000
End
    Frequencies[1/cm]           5
  2730.1      1598.0      1057.9      416.27      218.14
ZeroEnergy[kcal/mol]   -1.2196770344013361
 ElectronicLevels[1/cm]                     1
  0.0000000000000000        2.0000000000000000
End
!*********************************************
RRHO           !           99
Geometry[angstrom]           4
Cl   1.36560   0.01930  -0.00617
H   -0.15606  -0.18147   0.64119
S   -1.44454  -0.05578  -0.01591
H   -1.40011   1.28119   0.07742
 Core  RigidRotor
          SymmetryFactor   0.50000000000000000
End
    Frequencies[1/cm]           5
  2729.6      1537.3      1044.1      418.64      222.94
ZeroEnergy[kcal/mol]   -1.2664584242452357
 ElectronicLevels[1/cm]                     1
  0.0000000000000000        2.0000000000000000
End
!*********************************************
RRHO           !          100
Geometry[angstrom]           4
Cl   1.36537   0.01926  -0.00599
H   -0.14168  -0.17985   0.63466
S   -1.44475  -0.05578  -0.01589
H   -1.40002   1.28126   0.07723
 Core  RigidRotor
          SymmetryFactor   0.50000000000000000
End
    Frequencies[1/cm]           5
  2729.1      1482.5      1032.3      423.97      228.94
ZeroEnergy[kcal/mol]   -1.3085493681677438
 ElectronicLevels[1/cm]                     1
  0.0000000000000000        2.0000000000000000
End
!*********************************************
RRHO           !          101
Geometry[angstrom]           4
S    0.00000   0.00000   0.00000
H    0.00000   0.00000   1.34174
H    1.48190   0.00000  -0.02582
Cl   2.55774  -1.20674   0.18741
 Core  RigidRotor
          SymmetryFactor   0.50000000000000000
End
```

```
   Frequencies[1/cm]           5
  2728.7      1427.7      1014.0      422.35      232.63
ZeroEnergy[kcal/mol]   -1.3981992624439694
 ElectronicLevels[1/cm]                     1
  0.0000000000000000        2.0000000000000000
End
!**********************************************
RRHO           !          102
Geometry[angstrom]           4
Cl   1.36495   0.01917  -0.00564
H   -0.11227  -0.17684   0.62311
S   -1.44523  -0.05579  -0.01590
H   -1.39980   1.28137   0.07693
 Core  RigidRotor
          SymmetryFactor   0.50000000000000000
End
   Frequencies[1/cm]           5
  2728.3      1375.8      995.55      420.71      235.90
ZeroEnergy[kcal/mol]   -1.5096615201384820
 ElectronicLevels[1/cm]                     1
  0.0000000000000000        2.0000000000000000
End
!**********************************************
RRHO           !          103
Geometry[angstrom]           4
Cl   1.36477   0.01913  -0.00547
H   -0.09731  -0.17545   0.61803
S   -1.44550  -0.05579  -0.01592
H   -1.39967   1.28141   0.07682
 Core  RigidRotor
          SymmetryFactor   0.50000000000000000
End
   Frequencies[1/cm]           5
  2727.8      1337.9      981.72      424.41      241.57
ZeroEnergy[kcal/mol]   -1.6083262095437896
 ElectronicLevels[1/cm]                     1
  0.0000000000000000        2.0000000000000000
End
!**********************************************
RRHO           !          104
Geometry[angstrom]           4
Cl   1.36459   0.01909  -0.00531
H   -0.08224  -0.17411   0.61327
S   -1.44579  -0.05579  -0.01595
H   -1.39952   1.28145   0.07671
 Core  RigidRotor
          SymmetryFactor   0.50000000000000000
End
   Frequencies[1/cm]           5
  2727.5      1299.2      964.92      424.74      245.39
ZeroEnergy[kcal/mol]   -1.7513611790346379
 ElectronicLevels[1/cm]                     1
  0.0000000000000000        2.0000000000000000
End
!**********************************************
RRHO           !          105
```

```
Geometry[angstrom]           4
Cl   1.36443   0.01905  -0.00515
H   -0.06706  -0.17283   0.60886
S   -1.44609  -0.05579  -0.01598
H   -1.39937   1.28148   0.07662
 Core  RigidRotor
          SymmetryFactor   0.50000000000000000
End
    Frequencies[1/cm]           5
  2727.2      1264.2      947.82      424.63      248.54
ZeroEnergy[kcal/mol]   -1.9223692408117470
 ElectronicLevels[1/cm]                     1
  0.0000000000000000        2.0000000000000000
End
!*********************************************
RRHO           !          106
Geometry[angstrom]           4
Cl   1.36428   0.01902  -0.00499
H   -0.05180  -0.17160   0.60478
S   -1.44642  -0.05578  -0.01602
H   -1.39920   1.28150   0.07652
 Core  RigidRotor
          SymmetryFactor   0.50000000000000000
End
    Frequencies[1/cm]           5
  2726.9      1232.5      930.41      424.04      250.93
ZeroEnergy[kcal/mol]   -2.1221223636140656
 ElectronicLevels[1/cm]                     1
  0.0000000000000000        2.0000000000000000
End
!*********************************************
RRHO           !          107
Geometry[angstrom]           4
Cl   1.36415   0.01898  -0.00484
H   -0.03646  -0.17044   0.60103
S   -1.44676  -0.05578  -0.01606
H   -1.39902   1.28152   0.07644
 Core  RigidRotor
          SymmetryFactor   0.50000000000000000
End
    Frequencies[1/cm]           5
  2726.6      1204.1      912.74      423.02      252.55
ZeroEnergy[kcal/mol]   -2.3381561325375895
 ElectronicLevels[1/cm]                     1
  0.0000000000000000        2.0000000000000000
End
!*********************************************
RRHO           !          108
Geometry[angstrom]           4
Cl   1.36403   0.01895  -0.00469
H   -0.02104  -0.16934   0.59760
S   -1.44712  -0.05578  -0.01611
H   -1.39884   1.28153   0.07636
 Core  RigidRotor
          SymmetryFactor   0.50000000000000000
End
```

```
    Frequencies[1/cm]           5
  2726.4      1179.1      894.84      421.60      253.56
ZeroEnergy[kcal/mol]   -2.5886382283970186
 ElectronicLevels[1/cm]                     1
  0.0000000000000000        2.0000000000000000
End
!*********************************************
RRHO           !          109
Geometry[angstrom]           4
Cl   1.36393   0.01891  -0.00455
H   -0.00556  -0.16831   0.59449
S   -1.44751  -0.05578  -0.01617
H   -1.39864   1.28154   0.07628
 Core  RigidRotor
          SymmetryFactor   0.50000000000000000
End
    Frequencies[1/cm]           5
  2726.2      1158.1      876.80      419.86      254.22
ZeroEnergy[kcal/mol]   -2.8595888902462985
 ElectronicLevels[1/cm]                     1
  0.0000000000000000        2.0000000000000000
End
!*********************************************
RRHO           !          110
Geometry[angstrom]           4
Cl   1.36384   0.01888  -0.00441
H    0.00998  -0.16735   0.59173
S   -1.44791  -0.05577  -0.01623
H   -1.39844   1.28154   0.07621
 Core  RigidRotor
          SymmetryFactor   0.50000000000000000
End
    Frequencies[1/cm]           5
  2726.0      1142.0      858.69      417.86      254.79
ZeroEnergy[kcal/mol]   -3.1430175373966329
 ElectronicLevels[1/cm]                     1
  0.0000000000000000        2.0000000000000000
End

!*********************************************
!  Tunneling    Eckart
!    ImaginaryFrequency[1/cm]    1076.56
!    WellDepth[kcal/mol]           7.35
!    WellDepth[kcal/mol]           12.65
!    End

      Tunneling    Read
   CutoffEnergy[1/cm]       3500
   ImaginaryFrequency[1/cm]      1076.5599999999999
   File imactint.dat
   End
!*********************************************
End
 End
```

VRC-TST reactive flux: flux_lr_so9.dat

0	7388.88
13.9008	16396
28.4966	30980.7
43.8222	52764.1
59.9141	83468.9
76.8106	125276
94.552	180699
113.18	252106
132.74	342081
153.278	452744
174.843	580815
197.486	725672
221.261	889861
246.224	1.08211e+06
272.436	1.30653e+06
299.959	1.56775e+06
328.858	1.87055e+06
359.201	2.21851e+06
391.062	2.62067e+06
424.516	3.08577e+06
459.643	3.62356e+06
496.526	4.2451e+06
535.253	4.96392e+06
575.916	5.79245e+06
618.613	6.7453e+06
663.444	7.84349e+06
710.517	9.08811e+06
759.944	1.05128e+07
811.842	1.21388e+07
866.335	1.3987e+07
923.552	1.60929e+07
983.631	1.84841e+07
1046.71	2.12299e+07
1112.95	2.43237e+07
1182.5	2.78659e+07
1255.52	3.14448e+07
1332.2	3.48072e+07
1412.71	3.8266e+07
1497.25	4.19167e+07
1586.01	4.56694e+07
1679.21	4.96416e+07
1777.07	5.3989e+07
1879.83	5.86709e+07
1987.72	6.37684e+07
2101.01	6.9319e+07
2219.96	7.53693e+07
2344.86	8.20702e+07
2476	8.95202e+07
2613.7	9.78191e+07

2758.29	1.07099e+08
2910.1	1.17503e+08
3069.51	1.29187e+08
3236.88	1.42265e+08
3412.63	1.56895e+08
3597.16	1.73316e+08
3790.92	1.91763e+08
3994.37	2.12575e+08
4207.99	2.36086e+08
4432.29	2.62665e+08
4667.8	2.92709e+08
4915.09	3.26624e+08
5174.75	3.6492e+08
5447.39	4.08341e+08
5733.66	4.57709e+08
6034.24	5.13803e+08
6349.85	5.77476e+08
6681.25	6.49699e+08
7029.21	7.31552e+08
7394.57	8.24541e+08
7778.2	9.30513e+08
8181.01	1.05119e+09
8603.96	1.18833e+09
9048.06	1.34386e+09
9514.36	1.52014e+09
10004	1.72116e+09
10518.1	1.94994e+09
11057.9	2.20985e+09
11624.7	2.50463e+09
12219.8	2.83886e+09
12844.7	3.21886e+09
13500.8	3.65113e+09
14189.8	4.1418e+09
14913.2	4.69888e+09
15672.7	5.32929e+09
16470.3	6.04446e+09
17307.7	6.85799e+09
18187	7.78227e+09
19110.2	8.82912e+09
20079.6	1.00109e+10
21097.5	1.13532e+10
22166.3	1.28807e+10
23288.5	1.4614e+10
24466.8	1.6568e+10
25704.1	1.87764e+10
27003.2	2.1293e+10
28367.2	2.41533e+10
29799.5	2.73799e+10
31303.4	3.10112e+10
32882.5	3.51457e+10
34540.5	3.98525e+10
36281.4	4.51663e+10
38109.4	5.11387e+10
40028.7	5.79183e+10
42044.1	6.56466e+10
44160.2	7.43746e+10

46382.1	8.41946e+10
48715.1	9.5299e+10
51164.8	1.07962e+11
53736.9	1.22272e+11
56437.6	1.38397e+11
59273.4	1.56582e+11
62251	1.77296e+11
65377.5	2.00726e+11
68660.2	2.27171e+11
72107.1	2.56942e+11
75726.4	2.90783e+11
79526.6	3.29108e+11
83516.9	3.72439e+11
87706.6	4.2114e+11
92105.8	4.76384e+11
96725	5.3903e+11
101575	6.09961e+11
106668	6.89571e+11
112015	7.79714e+11
117630	8.82053e+11
123525	9.98059e+11
129715	1.12812e+12
136215	1.27516e+12
143040	1.44227e+12
150206	1.63185e+12
157730	1.84422e+12
165630	2.08402e+12
173925	2.35681e+12
182636	2.6664e+12
191781	3.013e+12
201384	3.40401e+12
211467	3.84921e+12
222055	4.35451e+12
233171	4.91991e+12
244844	5.55743e+12
257100	6.28388e+12
269969	7.10821e+12
283481	8.03019e+12
297669	9.06952e+12
312566	1.02547e+13
328209	1.1599e+13
344633	1.3102e+13
361879	1.47962e+13
379986	1.67296e+13
399000	1.89212e+13
418964	2.13706e+13
439926	2.41324e+13
461936	2.72858e+13
485046	3.08578e+13
509313	3.48489e+13
534792	3.93505e+13
561546	4.44934e+13
589637	5.03142e+13
619133	5.68159e+13
650103	6.41536e+13
682622	7.25403e+13

716767	8.20236e+13
752620	9.26142e+13
790264	1.04574e+14
829792	1.1825e+14
871295	1.33698e+14
914874	1.50946e+14
960631	1.70418e+14
1.00868e+06	1.92647e+14
1.05912e+06	2.17698e+14
1.11209e+06	2.45654e+14
1.16771e+06	2.7739e+14
1.22611e+06	3.13925e+14
1.28743e+06	3.55282e+14
1.35182e+06	4.0151e+14
1.41942e+06	4.53217e+14
1.49041e+06	5.11104e+14
1.56494e+06	5.75398e+14
1.6432e+06	6.46402e+14

imactint.dat

```
-2875 32.172892
-2874 32.144386
-2873 32.118688
-2872 32.092188
-2871 32.065239
-2870 32.036054
-2869 32.009566
-2868 31.984226
-2867 31.963638
-2866 31.938160
-2865 31.916402
-2864 31.886419
-2863 31.864216
-2862 31.834232
-2861 31.807367
-2860 31.784998
-2859 31.757309
-2858 31.735833
-2857 31.705800
-2856 31.683633
-2855 31.654454
-2854 31.630251
-2853 31.601701
-2852 31.577348
-2851 31.554527
-2850 31.526769
-2849 31.502783
-2848 31.474426
-2847 31.447290
-2846 31.421098
-2845 31.396412
-2844 31.372141
-2843 31.345522
-2842 31.322525
-2841 31.302251
-2840 31.276827
-2839 31.250861
-2838 31.228395
-2837 31.207595
-2836 31.180451
-2835 31.154817
-2834 31.131917
-2833 31.106779
-2832 31.082240
-2831 31.057680
-2830 31.037641
-2829 31.011160
-2828 30.991041
-2827 30.962399
-2826 30.939063
-2825 30.912562
-2824 30.886591
-2823 30.862888
-2822 30.840249
```

-2821 30.814895
-2820 30.790254
-2819 30.767269
-2818 30.741040
-2817 30.717467
-2816 30.691105
-2815 30.666709
-2814 30.647240
-2813 30.626321
-2812 30.605642
-2811 30.581359
-2810 30.557578
-2809 30.535737
-2808 30.512270
-2807 30.487973
-2806 30.463688
-2805 30.444882
-2804 30.422319
-2803 30.397638
-2802 30.374334
-2801 30.347895
-2800 30.326366
-2799 30.302150
-2798 30.278589
-2797 30.254099
-2796 30.234267
-2795 30.209238
-2794 30.185465
-2793 30.163585
-2792 30.139506
-2791 30.116732
-2790 30.092618
-2789 30.069756
-2788 30.045176
-2787 30.022612
-2786 30.003770
-2785 29.983299
-2784 29.962733
-2783 29.941681
-2782 29.920048
-2781 29.894587
-2780 29.875438
-2779 29.852515
-2778 29.828554
-2777 29.806157
-2776 29.786344
-2775 29.762704
-2774 29.745787
-2773 29.722995
-2772 29.698737
-2771 29.674457
-2770 29.653691
-2769 29.627023
-2768 29.607443
-2767 29.581429
-2766 29.559948

-2765	29.537365
-2764	29.514936
-2763	29.495493
-2762	29.469766
-2761	29.450063
-2760	29.425223
-2759	29.401833
-2758	29.378620
-2757	29.355503
-2756	29.337174
-2755	29.318510
-2754	29.299966
-2753	29.278934
-2752	29.256950
-2751	29.236213
-2750	29.216799
-2749	29.193063
-2748	29.173583
-2747	29.147962
-2746	29.130688
-2745	29.106846
-2744	29.083019
-2743	29.062919
-2742	29.044209
-2741	29.020985
-2740	28.998396
-2739	28.979968
-2738	28.957293
-2737	28.932190
-2736	28.910018
-2735	28.889525
-2734	28.864858
-2733	28.843087
-2732	28.821967
-2731	28.802803
-2730	28.780803
-2729	28.759522
-2728	28.739956
-2727	28.714447
-2726	28.697801
-2725	28.676523
-2724	28.655167
-2723	28.635301
-2722	28.615879
-2721	28.593106
-2720	28.570175
-2719	28.553126
-2718	28.534293
-2717	28.511840
-2716	28.491209
-2715	28.470952
-2714	28.449830
-2713	28.429845
-2712	28.405845
-2711	28.382342
-2710	28.363713

-2709 28.339898
-2708 28.321431
-2707 28.300067
-2706 28.281040
-2705 28.259796
-2704 28.238291
-2703 28.219018
-2702 28.196277
-2701 28.175199
-2700 28.152172
-2699 28.133237
-2698 28.111698
-2697 28.088840
-2696 28.070786
-2695 28.051400
-2694 28.030833
-2693 28.011452
-2692 27.993084
-2691 27.975434
-2690 27.954129
-2689 27.935007
-2688 27.915113
-2687 27.892860
-2686 27.876168
-2685 27.852433
-2684 27.834519
-2683 27.811027
-2682 27.789341
-2681 27.768723
-2680 27.745887
-2679 27.726422
-2678 27.708117
-2677 27.689771
-2676 27.670306
-2675 27.649425
-2674 27.628545
-2673 27.610209
-2672 27.585743
-2671 27.567839
-2670 27.546413
-2669 27.522458
-2668 27.501881
-2667 27.483798
-2666 27.464347
-2665 27.446307
-2664 27.424592
-2663 27.410351
-2662 27.391612
-2661 27.374849
-2660 27.356325
-2659 27.336973
-2658 27.318176
-2657 27.296199
-2656 27.275311
-2655 27.255231
-2654 27.233024

-2653	27.213237
-2652	27.194593
-2651	27.172101
-2650	27.155057
-2649	27.133065
-2648	27.112923
-2647	27.095044
-2646	27.075579
-2645	27.056870
-2644	27.037590
-2643	27.016194
-2642	26.997152
-2641	26.975107
-2640	26.955235
-2639	26.935942
-2638	26.914018
-2637	26.894172
-2636	26.871482
-2635	26.856057
-2634	26.837794
-2633	26.818905
-2632	26.800780
-2631	26.784601
-2630	26.767323
-2629	26.747836
-2628	26.729790
-2627	26.710679
-2626	26.695794
-2625	26.672839
-2624	26.656510
-2623	26.633420
-2622	26.613522
-2621	26.594964
-2620	26.575866
-2619	26.552992
-2618	26.532812
-2617	26.516892
-2616	26.495857
-2615	26.474373
-2614	26.456744
-2613	26.438374
-2612	26.421919
-2611	26.401512
-2610	26.381900
-2609	26.364038
-2608	26.343266
-2607	26.323355
-2606	26.301983
-2605	26.283098
-2604	26.265411
-2603	26.247338
-2602	26.229296
-2601	26.209740
-2600	26.193718
-2599	26.173448
-2598	26.154372

-2597 26.135760

-2596 26.117317

-2595 26.100206

-2594 26.084155

-2593 26.066202

-2592 26.046992

-2591 26.028081

-2590 26.011874

-2589 25.990792

-2588 25.973088

-2587 25.955503

-2586 25.934422

-2585 25.912137

-2584 25.892489

-2583 25.877743

-2582 25.856952

-2581 25.836471

-2580 25.816828

-2579 25.796521

-2578 25.777820

-2577 25.761102

-2576 25.742007

-2575 25.724817

-2574 25.705579

-2573 25.686810

-2572 25.672538

-2571 25.652978

-2570 25.637287

-2569 25.621224

-2568 25.600125

-2567 25.585647

-2566 25.565896

-2565 25.550772

-2564 25.531989

-2563 25.513569

-2562 25.491117

-2561 25.472392

-2560 25.455409

-2559 25.439699

-2558 25.423306

-2557 25.406257

-2556 25.387734

-2555 25.371461

-2554 25.352293

-2553 25.333878

-2552 25.318249

-2551 25.297547

-2550 25.280623

-2549 25.259323

-2548 25.243468

-2547 25.224711

-2546 25.206268

-2545 25.185867

-2544 25.167276

-2543 25.150233

-2542 25.131269

-2541 25.112211
-2540 25.091650
-2539 25.073806
-2538 25.060178
-2537 25.045928
-2536 25.031768
-2535 25.016247
-2534 25.000114
-2533 24.983706
-2532 24.965040
-2531 24.947943
-2530 24.933101
-2529 24.913352
-2528 24.898358
-2527 24.880428
-2526 24.860882
-2525 24.844788
-2524 24.825293
-2523 24.808756
-2522 24.791228
-2521 24.770740
-2520 24.751826
-2519 24.731990
-2518 24.716226
-2517 24.696927
-2516 24.680489
-2515 24.665633
-2514 24.648172
-2513 24.631479
-2512 24.615952
-2511 24.596863
-2510 24.579380
-2509 24.562130
-2508 24.542839
-2507 24.524217
-2506 24.508891
-2505 24.488475
-2504 24.476307
-2503 24.458139
-2502 24.440568
-2501 24.425288
-2500 24.409491
-2499 24.391587
-2498 24.375803
-2497 24.358029
-2496 24.340522
-2495 24.325032
-2494 24.310211
-2493 24.295070
-2492 24.278633
-2491 24.261762
-2490 24.246566
-2489 24.227329
-2488 24.213036
-2487 24.195891
-2486 24.176916

-2485	24.159733
-2484	24.142474
-2483	24.125301
-2482	24.107821
-2481	24.090631
-2480	24.075126
-2479	24.057809
-2478	24.040081
-2477	24.020920
-2476	24.004900
-2475	23.987438
-2474	23.968184
-2473	23.950343
-2472	23.932375
-2471	23.913977
-2470	23.897735
-2469	23.884205
-2468	23.870594
-2467	23.854932
-2466	23.841806
-2465	23.826624
-2464	23.811348
-2463	23.793962
-2462	23.776127
-2461	23.763192
-2460	23.744034
-2459	23.728297
-2458	23.713254
-2457	23.696827
-2456	23.680018
-2455	23.661242
-2454	23.643684
-2453	23.627758
-2452	23.611000
-2451	23.592615
-2450	23.576405
-2449	23.559533
-2448	23.540535
-2447	23.523238
-2446	23.508703
-2445	23.494304
-2444	23.477653
-2443	23.461710
-2442	23.445597
-2441	23.429383
-2440	23.412906
-2439	23.398354
-2438	23.379708
-2437	23.365198
-2436	23.345972
-2435	23.331343
-2434	23.310877
-2433	23.296759
-2432	23.281781
-2431	23.267150
-2430	23.251816

-2429 23.235793
-2428 23.220375
-2427 23.203115
-2426 23.189533
-2425 23.172607
-2424 23.155571
-2423 23.137305
-2422 23.120376
-2421 23.107811
-2420 23.093105
-2419 23.075695
-2418 23.060632
-2417 23.045503
-2416 23.029286
-2415 23.012683
-2414 22.997368
-2413 22.980655
-2412 22.965025
-2411 22.950001
-2410 22.934134
-2409 22.915542
-2408 22.899447
-2407 22.882609
-2406 22.865832
-2405 22.848124
-2404 22.834029
-2403 22.816551
-2402 22.798955
-2401 22.782969
-2400 22.765142
-2399 22.746786
-2398 22.730480
-2397 22.716491
-2396 22.701954
-2395 22.689951
-2394 22.675370
-2393 22.661685
-2392 22.644907
-2391 22.630609
-2390 22.618568
-2389 22.604072
-2388 22.586222
-2387 22.569334
-2386 22.554831
-2385 22.537898
-2384 22.521416
-2383 22.506066
-2382 22.491326
-2381 22.475824
-2380 22.457159
-2379 22.441550
-2378 22.424673
-2377 22.408995
-2376 22.392022
-2375 22.375078
-2374 22.358502

```
-2373 22.342959
-2372 22.326331
-2371 22.308048
-2370 22.292326
-2369 22.276300
-2368 22.260605
-2367 22.244603
-2366 22.229617
-2365 22.215445
-2364 22.198740
-2363 22.183206
-2362 22.166762
-2361 22.152276
-2360 22.136093
-2359 22.122976
-2358 22.108254
-2357 22.094024
-2356 22.078901
-2355 22.066151
-2354 22.046694
-2353 22.033190
-2352 22.017919
-2351 22.004375
-2350 21.986304
-2349 21.970381
-2348 21.952371
-2347 21.936824
-2346 21.921932
-2345 21.904891
-2344 21.889380
-2343 21.872688
-2342 21.856290
-2341 21.839742
-2340 21.824039
-2339 21.811528
-2338 21.794292
-2337 21.781822
-2336 21.764317
-2335 21.751732
-2334 21.733612
-2333 21.719159
-2332 21.702149
-2331 21.687484
-2330 21.670728
-2329 21.654000
-2328 21.638724
-2327 21.621609
-2326 21.608215
-2325 21.589584
-2324 21.572805
-2323 21.556500
-2322 21.544436
-2321 21.528157
-2320 21.515465
-2319 21.498552
-2318 21.485551
```

-2317 21.469739

-2316 21.452838

-2315 21.436917

-2314 21.423063

-2313 21.405522

-2312 21.390261

-2311 21.376801

-2310 21.366235

-2309 21.350548

-2308 21.335117

-2307 21.321043

-2306 21.304104

-2305 21.291309

-2304 21.273939

-2303 21.260983

-2302 21.243287

-2301 21.229872

-2300 21.210046

-2299 21.196363

-2298 21.181075

-2297 21.167135

-2296 21.147240

-2295 21.135345

-2294 21.115564

-2293 21.103331

-2292 21.085818

-2291 21.071101

-2290 21.050069

-2289 21.035091

-2288 21.021299

-2287 21.004571

-2286 20.988212

-2285 20.968717

-2284 20.955001

-2283 20.940659

-2282 20.927346

-2281 20.914549

-2280 20.903626

-2279 20.888438

-2278 20.877173

-2277 20.860935

-2276 20.845527

-2275 20.830390

-2274 20.818863

-2273 20.803408

-2272 20.787097

-2271 20.771336

-2270 20.756996

-2269 20.743483

-2268 20.728980

-2267 20.710661

-2266 20.696003

-2265 20.682193

-2264 20.665110

-2263 20.648968

-2262 20.634021

-2261 20.615251

-2260 20.600637

-2259 20.588166

-2258 20.571359

-2257 20.554945

-2256 20.537477

-2255 20.521801

-2254 20.506319

-2253 20.489488

-2252 20.474910

-2251 20.461894

-2250 20.446605

-2249 20.432074

-2248 20.419314

-2247 20.402760

-2246 20.386588

-2245 20.376584

-2244 20.362990

-2243 20.351218

-2242 20.333900

-2241 20.321699

-2240 20.305925

-2239 20.293523

-2238 20.277295

-2237 20.267222

-2236 20.250626

-2235 20.233139

-2234 20.218735

-2233 20.203558

-2232 20.188944

-2231 20.173722

-2230 20.161108

-2229 20.143681

-2228 20.130692

-2227 20.112551

-2226 20.098010

-2225 20.084202

-2224 20.069137

-2223 20.049788

-2222 20.036211

-2221 20.018657

-2220 20.003559

-2219 19.989737

-2218 19.976652

-2217 19.963711

-2216 19.949433

-2215 19.934983

-2214 19.923316

-2213 19.906359

-2212 19.892682

-2211 19.875218

-2210 19.863670

-2209 19.847720

-2208 19.834017

-2207 19.815028

-2206 19.803407

-2205 19.783414
-2204 19.775681
-2203 19.758434
-2202 19.744383
-2201 19.734142
-2200 19.716817
-2199 19.703802
-2198 19.688922
-2197 19.673491
-2196 19.660013
-2195 19.646299
-2194 19.632882
-2193 19.616578
-2192 19.600892
-2191 19.583530
-2190 19.570623
-2189 19.554146
-2188 19.537668
-2187 19.527645
-2186 19.514611
-2185 19.499674
-2184 19.487006
-2183 19.470750
-2182 19.457916
-2181 19.442689
-2180 19.432041
-2179 19.416176
-2178 19.401822
-2177 19.387471
-2176 19.370570
-2175 19.356176
-2174 19.343625
-2173 19.326824
-2172 19.309743
-2171 19.297712
-2170 19.284380
-2169 19.269686
-2168 19.250124
-2167 19.237581
-2166 19.222370
-2165 19.205757
-2164 19.193382
-2163 19.179644
-2162 19.163833
-2161 19.150886
-2160 19.136565
-2159 19.123390
-2158 19.111488
-2157 19.094909
-2156 19.080398
-2155 19.066006
-2154 19.054300
-2153 19.042794
-2152 19.030585
-2151 19.016267
-2150 19.003026

```
-2149 18.991759
-2148 18.978025
-2147 18.965091
-2146 18.948517
-2145 18.933431
-2144 18.922983
-2143 18.905130
-2142 18.895169
-2141 18.876440
-2140 18.859481
-2139 18.848099
-2138 18.832823
-2137 18.816946
-2136 18.801751
-2135 18.788134
-2134 18.775029
-2133 18.757036
-2132 18.745928
-2131 18.729539
-2130 18.716933
-2129 18.700343
-2128 18.685871
-2127 18.670673
-2126 18.657432
-2125 18.639586
-2124 18.624677
-2123 18.608907
-2122 18.595855
-2121 18.581915
-2120 18.572003
-2119 18.560687
-2118 18.552262
-2117 18.539694
-2116 18.525859
-2115 18.512097
-2114 18.502876
-2113 18.488194
-2112 18.473749
-2111 18.462016
-2110 18.447477
-2109 18.433036
-2108 18.420691
-2107 18.406119
-2106 18.393164
-2105 18.376559
-2104 18.365595
-2103 18.348714
-2102 18.337838
-2101 18.323066
-2100 18.309873
-2099 18.294646
-2098 18.281750
-2097 18.265956
-2096 18.248764
-2095 18.235223
-2094 18.222250
```

```
-2093 18.206783
-2092 18.191382
-2091 18.177848
-2090 18.161083
-2089 18.147339
-2088 18.132295
-2087 18.116989
-2086 18.102373
-2085 18.087447
-2084 18.074058
-2083 18.061938
-2082 18.049884
-2081 18.036460
-2080 18.024215
-2079 18.015727
-2078 18.000887
-2077 17.991869
-2076 17.980116
-2075 17.965911
-2074 17.952100
-2073 17.938544
-2072 17.924783
-2071 17.915124
-2070 17.898389
-2069 17.884713
-2068 17.874379
-2067 17.857896
-2066 17.845261
-2065 17.828933
-2064 17.818110
-2063 17.803727
-2062 17.790935
-2061 17.775973
-2060 17.761665
-2059 17.748162
-2058 17.732036
-2057 17.718797
-2056 17.705624
-2055 17.687298
-2054 17.674679
-2053 17.662153
-2052 17.645539
-2051 17.630312
-2050 17.616065
-2049 17.606163
-2048 17.593800
-2047 17.581241
-2046 17.569358
-2045 17.556062
-2044 17.541224
-2043 17.528807
-2042 17.516479
-2041 17.502210
-2040 17.488560
-2039 17.476195
-2038 17.465641
```

-2037	17.453735
-2036	17.443715
-2035	17.427198
-2034	17.417781
-2033	17.406874
-2032	17.390396
-2031	17.380359
-2030	17.367210
-2029	17.354884
-2028	17.339073
-2027	17.329138
-2026	17.312609
-2025	17.299348
-2024	17.288121
-2023	17.272691
-2022	17.259452
-2021	17.244256
-2020	17.234918
-2019	17.218547
-2018	17.205711
-2017	17.191351
-2016	17.175417
-2015	17.163689
-2014	17.148844
-2013	17.134587
-2012	17.119376
-2011	17.108521
-2010	17.096197
-2009	17.082877
-2008	17.071727
-2007	17.062104
-2006	17.048360
-2005	17.034446
-2004	17.021661
-2003	17.007770
-2002	16.996067
-2001	16.983561
-2000	16.968823
-1999	16.955141
-1998	16.942545
-1997	16.926972
-1996	16.915974
-1995	16.900698
-1994	16.888038
-1993	16.876457
-1992	16.867509
-1991	16.852086
-1990	16.842777
-1989	16.830369
-1988	16.815002
-1987	16.804066
-1986	16.790458
-1985	16.776813
-1984	16.765588
-1983	16.753767
-1982	16.738237

-1981	16.726181
-1980	16.711156
-1979	16.698792
-1978	16.684942
-1977	16.672208
-1976	16.658225
-1975	16.644835
-1974	16.633651
-1973	16.622502
-1972	16.611316
-1971	16.600769
-1970	16.589134
-1969	16.576810
-1968	16.561992
-1967	16.551177
-1966	16.537284
-1965	16.524440
-1964	16.510698
-1963	16.499653
-1962	16.484739
-1961	16.470870
-1960	16.459724
-1959	16.447827
-1958	16.433781
-1957	16.422515
-1956	16.407339
-1955	16.394932
-1954	16.381253
-1953	16.365427
-1952	16.353156
-1951	16.339453
-1950	16.324629
-1949	16.314353
-1948	16.305243
-1947	16.291905
-1946	16.281863
-1945	16.266988
-1944	16.254118
-1943	16.243615
-1942	16.231816
-1941	16.218786
-1940	16.207210
-1939	16.193811
-1938	16.181674
-1937	16.167326
-1936	16.153936
-1935	16.144688
-1934	16.132963
-1933	16.123250
-1932	16.110212
-1931	16.100937
-1930	16.087339
-1929	16.075372
-1928	16.064479
-1927	16.052057
-1926	16.038630

-1925	16.024284
-1924	16.012789
-1923	15.999466
-1922	15.990777
-1921	15.976491
-1920	15.962313
-1919	15.953845
-1918	15.938840
-1917	15.926749
-1916	15.914536
-1915	15.899259
-1914	15.884212
-1913	15.873962
-1912	15.861967
-1911	15.847215
-1910	15.832272
-1909	15.820507
-1908	15.807191
-1907	15.794691
-1906	15.777332
-1905	15.771376
-1904	15.759595
-1903	15.747245
-1902	15.738323
-1901	15.724317
-1900	15.713059
-1899	15.701417
-1898	15.688942
-1897	15.675548
-1896	15.663023
-1895	15.653566
-1894	15.643607
-1893	15.631832
-1892	15.621522
-1891	15.612275
-1890	15.599721
-1889	15.587533
-1888	15.578008
-1887	15.566104
-1886	15.552658
-1885	15.542957
-1884	15.531337
-1883	15.516089
-1882	15.506721
-1881	15.492247
-1880	15.481098
-1879	15.466969
-1878	15.455115
-1877	15.442876
-1876	15.433299
-1875	15.418526
-1874	15.407015
-1873	15.392826
-1872	15.380447
-1871	15.366742
-1870	15.354722

-1869	15.344312
-1868	15.330266
-1867	15.319247
-1866	15.304574
-1865	15.292866
-1864	15.278665
-1863	15.268741
-1862	15.253901
-1861	15.239938
-1860	15.229872
-1859	15.217797
-1858	15.206885
-1857	15.195800
-1856	15.184195
-1855	15.171357
-1854	15.162258
-1853	15.153407
-1852	15.142342
-1851	15.133389
-1850	15.122855
-1849	15.111404
-1848	15.101893
-1847	15.089607
-1846	15.079541
-1845	15.067504
-1844	15.055141
-1843	15.044674
-1842	15.032189
-1841	15.018834
-1840	15.007214
-1839	14.995620
-1838	14.983083
-1837	14.973895
-1836	14.959380
-1835	14.947695
-1834	14.934593
-1833	14.922552
-1832	14.910901
-1831	14.898482
-1830	14.888404
-1829	14.873458
-1828	14.860793
-1827	14.847797
-1826	14.837163
-1825	14.823843
-1824	14.810077
-1823	14.799456
-1822	14.785839
-1821	14.773391
-1820	14.760042
-1819	14.748099
-1818	14.735132
-1817	14.722289
-1816	14.709092
-1815	14.695690
-1814	14.681967

-1813	14.675267
-1812	14.667846
-1811	14.658605
-1810	14.649392
-1809	14.638689
-1808	14.628743
-1807	14.618983
-1806	14.609955
-1805	14.598863
-1804	14.587020
-1803	14.575043
-1802	14.564554
-1801	14.555156
-1800	14.543074
-1799	14.532934
-1798	14.519358
-1797	14.506780
-1796	14.495641
-1795	14.484058
-1794	14.473073
-1793	14.459030
-1792	14.447074
-1791	14.436385
-1790	14.425762
-1789	14.413440
-1788	14.398695
-1787	14.388599
-1786	14.377497
-1785	14.364645
-1784	14.352921
-1783	14.339072
-1782	14.327577
-1781	14.317169
-1780	14.301718
-1779	14.291294
-1778	14.278985
-1777	14.267202
-1776	14.254274
-1775	14.242507
-1774	14.228533
-1773	14.216366
-1772	14.202817
-1771	14.192989
-1770	14.182432
-1769	14.171824
-1768	14.161548
-1767	14.150223
-1766	14.138209
-1765	14.130264
-1764	14.122026
-1763	14.112412
-1762	14.100901
-1761	14.092166
-1760	14.081908
-1759	14.070834
-1758	14.060675

-1757	14.050239
-1756	14.038682
-1755	14.028271
-1754	14.017518
-1753	14.006464
-1752	13.994155
-1751	13.981730
-1750	13.974239
-1749	13.961078
-1748	13.950102
-1747	13.937905
-1746	13.926230
-1745	13.911803
-1744	13.900629
-1743	13.891073
-1742	13.879950
-1741	13.864854
-1740	13.856620
-1739	13.842636
-1738	13.832926
-1737	13.820531
-1736	13.807070
-1735	13.796059
-1734	13.783043
-1733	13.771440
-1732	13.760114
-1731	13.747072
-1730	13.734377
-1729	13.720052
-1728	13.712420
-1727	13.702193
-1726	13.692375
-1725	13.682774
-1724	13.670054
-1723	13.660384
-1722	13.649326
-1721	13.639153
-1720	13.625952
-1719	13.615758
-1718	13.606555
-1717	13.600913
-1716	13.590582
-1715	13.579394
-1714	13.571837
-1713	13.561742
-1712	13.549502
-1711	13.540442
-1710	13.530493
-1709	13.516078
-1708	13.505861
-1707	13.495468
-1706	13.485088
-1705	13.474659
-1704	13.463603
-1703	13.452389
-1702	13.442811

```
-1701 13.429791
-1700 13.419588
-1699 13.407051
-1698 13.397066
-1697 13.386846
-1696 13.372722
-1695 13.363621
-1694 13.348957
-1693 13.339852
-1692 13.327576
-1691 13.316893
-1690 13.303670
-1689 13.293832
-1688 13.282248
-1687 13.270040
-1686 13.258221
-1685 13.244764
-1684 13.234328
-1683 13.225648
-1682 13.217501
-1681 13.207303
-1680 13.197286
-1679 13.187130
-1678 13.176640
-1677 13.164834
-1676 13.156650
-1675 13.143522
-1674 13.132357
-1673 13.121348
-1672 13.111198
-1671 13.104708
-1670 13.095453
-1669 13.084363
-1668 13.074025
-1667 13.067142
-1666 13.058144
-1665 13.044204
-1664 13.037875
-1663 13.025639
-1662 13.016937
-1661 13.006730
-1660 12.993318
-1659 12.983979
-1658 12.972596
-1657 12.963177
-1656 12.951447
-1655 12.942115
-1654 12.931739
-1653 12.917869
-1652 12.908795
-1651 12.897248
-1650 12.887075
-1649 12.877726
-1648 12.866584
-1647 12.853945
-1646 12.842510
```

-1645	12.830795
-1644	12.819784
-1643	12.810049
-1642	12.796712
-1641	12.785774
-1640	12.773650
-1639	12.765268
-1638	12.756845
-1637	12.747506
-1636	12.738587
-1635	12.728620
-1634	12.720084
-1633	12.709903
-1632	12.700295
-1631	12.687687
-1630	12.679642
-1629	12.667323
-1628	12.659632
-1627	12.645550
-1626	12.636018
-1625	12.628738
-1624	12.620606
-1623	12.610263
-1622	12.601167
-1621	12.589717
-1620	12.581487
-1619	12.570308
-1618	12.560836
-1617	12.552623
-1616	12.540567
-1615	12.530131
-1614	12.522108
-1613	12.511021
-1612	12.500822
-1611	12.488517
-1610	12.481504
-1609	12.469512
-1608	12.458295
-1607	12.449032
-1606	12.437433
-1605	12.425860
-1604	12.415756
-1603	12.405589
-1602	12.393958
-1601	12.384866
-1600	12.374361
-1599	12.363216
-1598	12.350792
-1597	12.341310
-1596	12.328867
-1595	12.320781
-1594	12.312170
-1593	12.303007
-1592	12.295155
-1591	12.285614
-1590	12.274788

-1589	12.265898
-1588	12.256939
-1587	12.245709
-1586	12.236224
-1585	12.227968
-1584	12.216599
-1583	12.205008
-1582	12.196913
-1581	12.188632
-1580	12.181788
-1579	12.170299
-1578	12.163322
-1577	12.153962
-1576	12.142238
-1575	12.134128
-1574	12.123245
-1573	12.112394
-1572	12.104221
-1571	12.093704
-1570	12.081941
-1569	12.074351
-1568	12.064608
-1567	12.054630
-1566	12.043952
-1565	12.031780
-1564	12.022454
-1563	12.010358
-1562	12.001752
-1561	11.990164
-1560	11.980813
-1559	11.971633
-1558	11.960488
-1557	11.949803
-1556	11.938730
-1555	11.928688
-1554	11.917968
-1553	11.907479
-1552	11.897947
-1551	11.886586
-1550	11.875407
-1549	11.866021
-1548	11.857644
-1547	11.848553
-1546	11.842673
-1545	11.835232
-1544	11.827167
-1543	11.817178
-1542	11.809128
-1541	11.801362
-1540	11.791456
-1539	11.784002
-1538	11.773258
-1537	11.765275
-1536	11.753700
-1535	11.744544
-1534	11.737184

-1533	11.726834
-1532	11.716424
-1531	11.709242
-1530	11.699598
-1529	11.688999
-1528	11.677552
-1527	11.668482
-1526	11.657780
-1525	11.647779
-1524	11.639272
-1523	11.629024
-1522	11.616968
-1521	11.608482
-1520	11.596100
-1519	11.589434
-1518	11.576505
-1517	11.566728
-1516	11.558998
-1515	11.548975
-1514	11.538799
-1513	11.529875
-1512	11.519576
-1511	11.510130
-1510	11.500057
-1509	11.491420
-1508	11.481092
-1507	11.470895
-1506	11.460760
-1505	11.450589
-1504	11.440544
-1503	11.429890
-1502	11.419328
-1501	11.409618
-1500	11.402304
-1499	11.393942
-1498	11.385399
-1497	11.377928
-1496	11.369595
-1495	11.361344
-1494	11.354180
-1493	11.344307
-1492	11.334753
-1491	11.328187
-1490	11.317601
-1489	11.309667
-1488	11.300087
-1487	11.288774
-1486	11.278736
-1485	11.270920
-1484	11.260715
-1483	11.252415
-1482	11.242796
-1481	11.233286
-1480	11.225195
-1479	11.217218
-1478	11.206515

-1477	11.197740
-1476	11.189411
-1475	11.177193
-1474	11.167486
-1473	11.158871
-1472	11.150278
-1471	11.140081
-1470	11.129443
-1469	11.121492
-1468	11.111096
-1467	11.100870
-1466	11.091797
-1465	11.082479
-1464	11.071959
-1463	11.061973
-1462	11.054016
-1461	11.045241
-1460	11.036065
-1459	11.026164
-1458	11.017754
-1457	11.007776
-1456	10.997246
-1455	10.989463
-1454	10.982751
-1453	10.976372
-1452	10.967662
-1451	10.960185
-1450	10.952595
-1449	10.943534
-1448	10.935492
-1447	10.928704
-1446	10.918385
-1445	10.909193
-1444	10.900920
-1443	10.892604
-1442	10.885535
-1441	10.876093
-1440	10.865463
-1439	10.859449
-1438	10.849299
-1437	10.841552
-1436	10.829912
-1435	10.821784
-1434	10.813254
-1433	10.804809
-1432	10.793677
-1431	10.784624
-1430	10.776265
-1429	10.766552
-1428	10.756384
-1427	10.748338
-1426	10.741979
-1425	10.731539
-1424	10.723081
-1423	10.715365
-1422	10.704663

```
-1421 10.697795
-1420 10.687260
-1419 10.677741
-1418 10.670905
-1417 10.661836
-1416 10.653466
-1415 10.642679
-1414 10.634123
-1413 10.626009
-1412 10.616742
-1411 10.606320
-1410 10.598044
-1409 10.587717
-1408 10.579112
-1407 10.569388
-1406 10.558645
-1405 10.550921
-1404 10.544738
-1403 10.536774
-1402 10.529104
-1401 10.521849
-1400 10.513588
-1399 10.505696
-1398 10.498710
-1397 10.489403
-1396 10.481602
-1395 10.472500
-1394 10.465218
-1393 10.454747
-1392 10.449014
-1391 10.439087
-1390 10.429950
-1389 10.421428
-1388 10.410836
-1387 10.403352
-1386 10.393391
-1385 10.385011
-1384 10.376143
-1383 10.367620
-1382 10.359236
-1381 10.350812
-1380 10.341546
-1379 10.332630
-1378 10.321183
-1377 10.313706
-1376 10.304417
-1375 10.295111
-1374 10.285474
-1373 10.275921
-1372 10.267729
-1371 10.260013
-1370 10.250868
-1369 10.244015
-1368 10.234045
-1367 10.224283
-1366 10.216490
```

-1365	10.207808
-1364	10.200394
-1363	10.190744
-1362	10.181809
-1361	10.172324
-1360	10.163466
-1359	10.153914
-1358	10.143115
-1357	10.137730
-1356	10.131522
-1355	10.124118
-1354	10.116905
-1353	10.110287
-1352	10.101817
-1351	10.093898
-1350	10.085557
-1349	10.078219
-1348	10.070994
-1347	10.061586
-1346	10.055263
-1345	10.045270
-1344	10.038031
-1343	10.027269
-1342	10.021693
-1341	10.012789
-1340	10.002666
-1339	9.994978
-1338	9.985744
-1337	9.977803
-1336	9.968335
-1335	9.958049
-1334	9.949905
-1333	9.942717
-1332	9.935133
-1331	9.925115
-1330	9.914506
-1329	9.904782
-1328	9.896472
-1327	9.890064
-1326	9.879071
-1325	9.870012
-1324	9.862811
-1323	9.851857
-1322	9.843487
-1321	9.834552
-1320	9.826392
-1319	9.816686
-1318	9.807564
-1317	9.799747
-1316	9.789847
-1315	9.780522
-1314	9.772126
-1313	9.761367
-1312	9.752850
-1311	9.744255
-1310	9.734863

-1309	9.724446
-1308	9.715826
-1307	9.710056
-1306	9.702603
-1305	9.696053
-1304	9.687576
-1303	9.679134
-1302	9.672551
-1301	9.663545
-1300	9.656193
-1299	9.647872
-1298	9.638649
-1297	9.630790
-1296	9.624774
-1295	9.617130
-1294	9.609628
-1293	9.602154
-1292	9.592894
-1291	9.584856
-1290	9.577191
-1289	9.569742
-1288	9.560547
-1287	9.552886
-1286	9.545223
-1285	9.535998
-1284	9.529540
-1283	9.519308
-1282	9.512526
-1281	9.503431
-1280	9.494849
-1279	9.485069
-1278	9.478091
-1277	9.469577
-1276	9.460895
-1275	9.451139
-1274	9.443121
-1273	9.433071
-1272	9.425769
-1271	9.417180
-1270	9.408277
-1269	9.399005
-1268	9.388627
-1267	9.382510
-1266	9.372747
-1265	9.362605
-1264	9.355732
-1263	9.345760
-1262	9.337590
-1261	9.327525
-1260	9.317649
-1259	9.307370
-1258	9.302806
-1257	9.296462
-1256	9.289226
-1255	9.281250
-1254	9.273586

-1253 9.266976
-1252 9.257722
-1251 9.250512
-1250 9.242171
-1249 9.235946
-1248 9.226525
-1247 9.218129
-1246 9.209955
-1245 9.201732
-1244 9.194002
-1243 9.186365
-1242 9.178843
-1241 9.167023
-1240 9.160898
-1239 9.151091
-1238 9.142459
-1237 9.135112
-1236 9.126082
-1235 9.117352
-1234 9.107365
-1233 9.101393
-1232 9.092970
-1231 9.084179
-1230 9.074494
-1229 9.066877
-1228 9.055364
-1227 9.047953
-1226 9.039079
-1225 9.029540
-1224 9.020478
-1223 9.011748
-1222 9.003197
-1221 8.994736
-1220 8.985675
-1219 8.976792
-1218 8.968766
-1217 8.959996
-1216 8.950718
-1215 8.940328
-1214 8.932552
-1213 8.921884
-1212 8.914036
-1211 8.906746
-1210 8.898288
-1209 8.889727
-1208 8.879938
-1207 8.875382
-1206 8.869327
-1205 8.862626
-1204 8.856228
-1203 8.849682
-1202 8.842174
-1201 8.835157
-1200 8.829090
-1199 8.820471
-1198 8.813026

-1197	8.805285
-1196	8.798859
-1195	8.791986
-1194	8.781804
-1193	8.775432
-1192	8.767849
-1191	8.757962
-1190	8.749450
-1189	8.743475
-1188	8.735438
-1187	8.725552
-1186	8.718852
-1185	8.708513
-1184	8.702180
-1183	8.691963
-1182	8.686746
-1181	8.676720
-1180	8.668405
-1179	8.659920
-1178	8.652100
-1177	8.642125
-1176	8.634604
-1175	8.624995
-1174	8.617278
-1173	8.606914
-1172	8.600064
-1171	8.592162
-1170	8.581961
-1169	8.573534
-1168	8.564727
-1167	8.556034
-1166	8.548228
-1165	8.540356
-1164	8.529961
-1163	8.521726
-1162	8.512085
-1161	8.503890
-1160	8.494431
-1159	8.485500
-1158	8.476817
-1157	8.466781
-1156	8.461450
-1155	8.454755
-1154	8.448481
-1153	8.441260
-1152	8.434170
-1151	8.425601
-1150	8.419472
-1149	8.409328
-1148	8.402245
-1147	8.396388
-1146	8.388607
-1145	8.379785
-1144	8.372659
-1143	8.363856
-1142	8.353762

-1141 8.347302

-1140 8.338498

-1139 8.330299

-1138 8.320946

-1137 8.313523

-1136 8.306438

-1135 8.297282

-1134 8.289352

-1133 8.278997

-1132 8.271888

-1131 8.264139

-1130 8.253815

-1129 8.245475

-1128 8.238082

-1127 8.228095

-1126 8.218774

-1125 8.211410

-1124 8.203658

-1123 8.194329

-1122 8.185298

-1121 8.175961

-1120 8.167201

-1119 8.159422

-1118 8.148972

-1117 8.140255

-1116 8.133399

-1115 8.123293

-1114 8.115034

-1113 8.104471

-1112 8.098430

-1111 8.089350

-1110 8.082228

-1109 8.074179

-1108 8.065525

-1107 8.057222

-1106 8.051039

-1105 8.045595

-1104 8.039565

-1103 8.032647

-1102 8.027047

-1101 8.018877

-1100 8.012154

-1099 8.005767

-1098 7.999095

-1097 7.991800

-1096 7.983352

-1095 7.976575

-1094 7.969372

-1093 7.961447

-1092 7.953411

-1091 7.943983

-1090 7.937097

-1089 7.929721

-1088 7.922754

-1087 7.913618

-1086 7.905936

-1085 7.899147

-1084 7.889893

-1083 7.881196

-1082 7.872537

-1081 7.865438

-1080 7.857967

-1079 7.849669

-1078 7.839583

-1077 7.832368

-1076 7.821910

-1075 7.814304

-1074 7.807194

-1073 7.798302

-1072 7.789892

-1071 7.781137

-1070 7.773795

-1069 7.764212

-1068 7.757620

-1067 7.747615

-1066 7.740112

-1065 7.731900

-1064 7.722989

-1063 7.714151

-1062 7.704523

-1061 7.697208

-1060 7.687805

-1059 7.679621

-1058 7.670176

-1057 7.660134

-1056 7.653764

-1055 7.647012

-1054 7.641186

-1053 7.633265

-1052 7.626539

-1051 7.619389

-1050 7.611909

-1049 7.605522

-1048 7.596230

-1047 7.590443

-1046 7.583449

-1045 7.573199

-1044 7.567568

-1043 7.558563

-1042 7.550212

-1041 7.542217

-1040 7.533982

-1039 7.526356

-1038 7.519269

-1037 7.509440

-1036 7.502267

-1035 7.492416

-1034 7.487409

-1033 7.477566

-1032 7.469307

-1031 7.462123

-1030 7.451968

-1029 7.445093

-1028 7.437313

-1027 7.427805

-1026 7.418041

-1025 7.411401

-1024 7.402449

-1023 7.393031

-1022 7.383947

-1021 7.376840

-1020 7.368110

-1019 7.360065

-1018 7.351237

-1017 7.341002

-1016 7.334198

-1015 7.324439

-1014 7.316434

-1013 7.307937

-1012 7.297999

-1011 7.289455

-1010 7.280591

-1009 7.271527

-1008 7.263345

-1007 7.256508

-1006 7.248276

-1005 7.239789

-1004 7.234810

-1003 7.229516

-1002 7.223887

-1001 7.217305

-1000 7.211797

-999 7.204668

-998 7.198385

-997 7.191447

-996 7.184308

-995 7.176157

-994 7.170345

-993 7.163156

-992 7.154926

-991 7.148891

-990 7.140533

-989 7.134431

-988 7.127410

-987 7.119115

-986 7.110479

-985 7.103837

-984 7.094737

-983 7.088311

-982 7.078675

-981 7.071378

-980 7.063848

-979 7.054144

-978 7.046080

-977 7.039578

-976 7.031281

-975 7.023817

-974 7.016094

-973	7.006521
-972	6.997585
-971	6.989745
-970	6.982875
-969	6.973275
-968	6.965958
-967	6.958004
-966	6.949545
-965	6.940356
-964	6.931060
-963	6.924772
-962	6.917179
-961	6.906904
-960	6.899059
-959	6.891357
-958	6.882820
-957	6.873204
-956	6.865654
-955	6.856528
-954	6.847370
-953	6.839243
-952	6.834411
-951	6.828288
-950	6.822284
-949	6.813787
-948	6.806407
-947	6.799149
-946	6.793827
-945	6.784754
-944	6.777600
-943	6.771005
-942	6.764528
-941	6.754517
-940	6.747430
-939	6.738592
-938	6.732540
-937	6.724132
-936	6.717678
-935	6.709332
-934	6.700756
-933	6.692764
-932	6.685024
-931	6.677413
-930	6.668912
-929	6.658658
-928	6.651606
-927	6.643558
-926	6.636211
-925	6.627073
-924	6.618790
-923	6.611135
-922	6.603597
-921	6.595308
-920	6.586812
-919	6.578930
-918	6.570066

-917	6.559579
-916	6.552813
-915	6.545055
-914	6.537079
-913	6.526476
-912	6.519072
-911	6.511563
-910	6.501332
-909	6.492994
-908	6.485967
-907	6.477016
-906	6.467826
-905	6.458232
-904	6.450142
-903	6.439876
-902	6.432638
-901	6.425506
-900	6.418987
-899	6.414921
-898	6.409316
-897	6.403284
-896	6.397330
-895	6.391166
-894	6.385595
-893	6.377515
-892	6.372167
-891	6.364616
-890	6.356608
-889	6.350848
-888	6.342998
-887	6.336274
-886	6.328579
-885	6.322763
-884	6.314626
-883	6.307372
-882	6.300889
-881	6.292039
-880	6.285488
-879	6.275682
-878	6.270214
-877	6.261474
-876	6.252228
-875	6.244832
-874	6.238384
-873	6.230700
-872	6.221521
-871	6.215553
-870	6.206621
-869	6.198184
-868	6.190279
-867	6.182386
-866	6.173597
-865	6.165463
-864	6.157354
-863	6.150052
-862	6.142553

-861	6.133854
-860	6.125984
-859	6.117766
-858	6.108217
-857	6.101679
-856	6.092413
-855	6.083502
-854	6.076412
-853	6.067402
-852	6.058140
-851	6.049990
-850	6.040149
-849	6.035732
-848	6.030094
-847	6.024612
-846	6.017148
-845	6.009790
-844	6.002911
-843	5.996331
-842	5.989005
-841	5.981545
-840	5.974516
-839	5.965929
-838	5.959336
-837	5.953560
-836	5.943710
-835	5.935833
-834	5.930051
-833	5.919992
-832	5.914056
-831	5.905490
-830	5.897966
-829	5.890747
-828	5.880955
-827	5.874049
-826	5.865242
-825	5.857531
-824	5.849167
-823	5.842324
-822	5.832556
-821	5.826056
-820	5.818695
-819	5.809286
-818	5.800775
-817	5.793931
-816	5.785168
-815	5.775761
-814	5.767538
-813	5.759252
-812	5.752238
-811	5.743179
-810	5.736068
-809	5.727693
-808	5.719868
-807	5.711043
-806	5.703006

-805 5.694659
-804 5.685297
-803 5.677138
-802 5.668368
-801 5.659722
-800 5.651244
-799 5.642238
-798 5.632148
-797 5.627281
-796 5.620741
-795 5.613406
-794 5.608176
-793 5.603631
-792 5.597598
-791 5.591553
-790 5.585785
-789 5.579490
-788 5.573213
-787 5.566664
-786 5.560169
-785 5.552655
-784 5.545480
-783 5.539062
-782 5.531157
-781 5.525908
-780 5.517454
-779 5.510084
-778 5.503784
-777 5.495030
-776 5.488223
-775 5.480487
-774 5.473197
-773 5.466152
-772 5.458251
-771 5.451157
-770 5.442984
-769 5.435559
-768 5.426670
-767 5.420292
-766 5.412790
-765 5.404912
-764 5.396339
-763 5.388109
-762 5.380023
-761 5.373247
-760 5.364862
-759 5.356901
-758 5.349346
-757 5.340710
-756 5.331963
-755 5.324714
-754 5.317142
-753 5.309179
-752 5.300459
-751 5.293196
-750 5.283403

```
-749 5.275087
-748 5.267252
-747 5.257850
-746 5.250845
-745 5.245394
-744 5.239927
-743 5.233568
-742 5.227563
-741 5.218983
-740 5.212944
-739 5.206618
-738 5.199092
-737 5.191037
-736 5.184867
-735 5.176580
-734 5.170914
-733 5.161647
-732 5.154831
-731 5.148757
-730 5.138892
-729 5.131336
-728 5.124553
-727 5.118147
-726 5.109504
-725 5.101427
-724 5.093051
-723 5.084642
-722 5.076706
-721 5.070537
-720 5.061422
-719 5.053943
-718 5.044933
-717 5.037751
-716 5.030464
-715 5.020606
-714 5.013001
-713 5.005132
-712 4.996584
-711 4.988078
-710 4.981362
-709 4.971834
-708 4.965273
-707 4.956391
-706 4.948358
-705 4.939868
-704 4.931787
-703 4.922659
-702 4.914895
-701 4.906197
-700 4.898157
-699 4.889767
-698 4.881425
-697 4.872353
-696 4.862631
-695 4.856943
-694 4.850713
```

-693 4.844852
-692 4.837911
-691 4.830483
-690 4.822557
-689 4.817650
-688 4.811263
-687 4.806657
-686 4.800493
-685 4.793339
-684 4.788632
-683 4.780582
-682 4.774804
-681 4.767694
-680 4.760344
-679 4.754674
-678 4.748111
-677 4.740837
-676 4.732787
-675 4.726662
-674 4.718524
-673 4.710540
-672 4.703158
-671 4.697330
-670 4.689720
-669 4.682556
-668 4.673172
-667 4.667193
-666 4.660122
-665 4.650939
-664 4.643490
-663 4.636700
-662 4.627738
-661 4.620056
-660 4.612090
-659 4.604158
-658 4.597096
-657 4.588347
-656 4.581555
-655 4.573072
-654 4.565120
-653 4.556114
-652 4.549999
-651 4.540609
-650 4.533301
-649 4.525046
-648 4.517073
-647 4.507679
-646 4.499397
-645 4.492504
-644 4.486913
-643 4.481848
-642 4.474708
-641 4.468540
-640 4.462206
-639 4.454889
-638 4.448518

-637 4.441970
-636 4.433260
-635 4.426384
-634 4.419853
-633 4.411883
-632 4.405024
-631 4.398588
-630 4.390772
-629 4.382227
-628 4.375983
-627 4.367895
-626 4.358819
-625 4.352378
-624 4.344114
-623 4.336141
-622 4.328697
-621 4.320522
-620 4.312691
-619 4.305283
-618 4.297314
-617 4.288237
-616 4.280169
-615 4.273312
-614 4.264480
-613 4.257193
-612 4.248095
-611 4.241227
-610 4.233598
-609 4.223842
-608 4.215937
-607 4.208468
-606 4.201441
-605 4.192895
-604 4.184194
-603 4.175098
-602 4.167431
-601 4.159087
-600 4.151302
-599 4.142170
-598 4.133945
-597 4.125308
-596 4.115575
-595 4.111254
-594 4.105205
-593 4.099246
-592 4.091932
-591 4.085419
-590 4.078808
-589 4.074114
-588 4.068087
-587 4.062062
-586 4.056483
-585 4.050075
-584 4.043831
-583 4.037786
-582 4.031973

-581 4.024167

-580 4.017933

-579 4.012342

-578 4.004622

-577 3.997016

-576 3.989952

-575 3.984135

-574 3.977575

-573 3.968031

-572 3.962291

-571 3.954695

-570 3.945915

-569 3.939271

-568 3.932145

-567 3.924479

-566 3.917520

-565 3.909872

-564 3.902100

-563 3.894393

-562 3.885759

-561 3.879875

-560 3.871914

-559 3.863817

-558 3.856455

-557 3.848875

-556 3.841152

-555 3.831897

-554 3.825085

-553 3.817270

-552 3.809535

-551 3.801018

-550 3.793891

-549 3.785124

-548 3.777225

-547 3.768404

-546 3.759467

-545 3.752098

-544 3.746609

-543 3.741736

-542 3.735010

-541 3.729108

-540 3.723105

-539 3.716514

-538 3.709030

-537 3.702841

-536 3.696044

-535 3.688572

-534 3.682023

-533 3.675107

-532 3.667221

-531 3.659869

-530 3.652518

-529 3.645505

-528 3.638508

-527 3.630744

-526 3.622829

-525	3.614774
-524	3.607887
-523	3.599435
-522	3.592307
-521	3.584041
-520	3.577141
-519	3.568004
-518	3.560232
-517	3.553188
-516	3.545168
-515	3.538248
-514	3.529181
-513	3.520117
-512	3.512877
-511	3.505003
-510	3.497719
-509	3.489437
-508	3.480478
-507	3.472655
-506	3.464715
-505	3.457618
-504	3.449038
-503	3.440610
-502	3.432639
-501	3.424243
-500	3.415738
-499	3.407236
-498	3.397777
-497	3.390579
-496	3.384640
-495	3.378941
-494	3.375283
-493	3.370527
-492	3.365828
-491	3.359440
-490	3.354551
-489	3.348067
-488	3.342398
-487	3.336031
-486	3.329827
-485	3.323692
-484	3.315875
-483	3.310246
-482	3.302555
-481	3.296282
-480	3.288962
-479	3.283331
-478	3.275670
-477	3.268241
-476	3.262403
-475	3.253438
-474	3.246532
-473	3.239080
-472	3.231938
-471	3.225416
-470	3.216397

-469	3.210829
-468	3.202644
-467	3.194310
-466	3.186615
-465	3.179359
-464	3.172473
-463	3.164878
-462	3.157604
-461	3.150369
-460	3.141546
-459	3.132990
-458	3.126470
-457	3.118134
-456	3.111056
-455	3.102399
-454	3.095332
-453	3.086823
-452	3.078046
-451	3.069820
-450	3.064112
-449	3.058776
-448	3.052868
-447	3.047543
-446	3.041091
-445	3.034533
-444	3.027510
-443	3.021592
-442	3.014880
-441	3.007763
-440	3.001397
-439	2.994311
-438	2.986737
-437	2.980210
-436	2.972179
-435	2.965027
-434	2.958460
-433	2.950746
-432	2.943837
-431	2.935623
-430	2.928700
-429	2.920458
-428	2.912599
-427	2.905080
-426	2.898839
-425	2.889328
-424	2.883257
-423	2.873810
-422	2.867269
-421	2.859915
-420	2.850812
-419	2.844170
-418	2.836728
-417	2.827549
-416	2.820650
-415	2.811999
-414	2.804925

-413	2.796284
-412	2.788179
-411	2.780379
-410	2.771762
-409	2.763491
-408	2.755123
-407	2.747770
-406	2.741804
-405	2.734950
-404	2.727684
-403	2.720147
-402	2.714952
-401	2.711170
-400	2.706265
-399	2.700854
-398	2.695234
-397	2.690380
-396	2.684451
-395	2.677859
-394	2.672267
-393	2.665251
-392	2.658811
-391	2.653411
-390	2.646730
-389	2.639692
-388	2.632990
-387	2.625352
-386	2.619759
-385	2.612103
-384	2.603995
-383	2.597482
-382	2.590228
-381	2.583391
-380	2.575384
-379	2.567805
-378	2.561095
-377	2.552865
-376	2.546079
-375	2.537715
-374	2.530743
-373	2.523867
-372	2.515221
-371	2.506910
-370	2.500358
-369	2.492244
-368	2.484261
-367	2.476374
-366	2.469429
-365	2.461089
-364	2.454020
-363	2.445734
-362	2.438527
-361	2.429714
-360	2.421998
-359	2.413015
-358	2.404213

-357	2.399048
-356	2.393601
-355	2.388126
-354	2.381870
-353	2.376059
-352	2.369314
-351	2.363899
-350	2.356739
-349	2.349343
-348	2.343527
-347	2.336452
-346	2.328799
-345	2.321707
-344	2.314596
-343	2.307763
-342	2.300007
-341	2.292311
-340	2.285061
-339	2.277430
-338	2.269160
-337	2.262250
-336	2.253816
-335	2.246445
-334	2.238421
-333	2.230251
-332	2.221873
-331	2.215279
-330	2.209985
-329	2.203589
-328	2.198012
-327	2.190488
-326	2.184842
-325	2.177177
-324	2.170437
-323	2.163561
-322	2.157367
-321	2.150046
-320	2.142368
-319	2.135277
-318	2.127757
-317	2.120691
-316	2.113303
-315	2.104748
-314	2.096575
-313	2.093477
-312	2.088430
-311	2.082695
-310	2.077521
-309	2.071238
-308	2.065765
-307	2.058779
-306	2.052541
-305	2.046426
-304	2.039200
-303	2.032441
-302	2.026362

-301 2.018521
-300 2.012232
-299 2.004948
-298 1.997624
-297 1.990348
-296 1.982732
-295 1.975955
-294 1.968447
-293 1.960722
-292 1.953509
-291 1.945227
-290 1.937976
-289 1.930846
-288 1.922916
-287 1.915739
-286 1.907552
-285 1.900051
-284 1.892008
-283 1.884985
-282 1.877125
-281 1.869488
-280 1.861035
-279 1.852877
-278 1.845706
-277 1.837292
-276 1.829724
-275 1.821418
-274 1.812004
-273 1.807415
-272 1.802819
-271 1.796803
-270 1.790976
-269 1.784971
-268 1.778029
-267 1.771947
-266 1.765094
-265 1.758088
-264 1.750932
-263 1.744346
-262 1.735967
-261 1.729803
-260 1.724762
-259 1.719682
-258 1.713430
-257 1.707755
-256 1.701338
-255 1.695561
-254 1.688136
-253 1.682284
-252 1.676091
-251 1.668559
-250 1.662134
-249 1.655285
-248 1.647891
-247 1.641518
-246 1.634033

```
-245 1.626590
-244 1.620319
-243 1.613021
-242 1.605383
-241 1.598215
-240 1.590595
-239 1.583249
-238 1.575829
-237 1.568100
-236 1.560603
-235 1.553142
-234 1.544903
-233 1.536146
-232 1.531275
-231 1.526305
-230 1.520912
-229 1.515185
-228 1.509269
-227 1.502713
-226 1.496544
-225 1.490201
-224 1.484150
-223 1.476525
-222 1.469277
-221 1.463475
-220 1.455760
-219 1.449033
-218 1.442000
-217 1.433914
-216 1.427251
-215 1.419715
-214 1.411670
-213 1.404605
-212 1.396957
-211 1.389151
-210 1.382196
-209 1.374665
-208 1.366525
-207 1.358791
-206 1.350777
-205 1.343213
-204 1.335670
-203 1.327297
-202 1.318854
-201 1.310861
-200 1.305407
-199 1.299572
-198 1.293574
-197 1.286670
-196 1.279410
-195 1.272917
-194 1.269602
-193 1.265505
-192 1.260346
-191 1.255719
-190 1.249945
```

-189	1.244193
-188	1.238223
-187	1.232146
-186	1.226216
-185	1.219483
-184	1.212920
-183	1.206635
-182	1.199580
-181	1.193013
-180	1.186581
-179	1.179099
-178	1.171406
-177	1.165251
-176	1.157294
-175	1.150146
-174	1.142917
-173	1.135817
-172	1.127920
-171	1.120300
-170	1.113177
-169	1.105606
-168	1.097875
-167	1.090333
-166	1.082702
-165	1.074966
-164	1.067164
-163	1.059449
-162	1.051534
-161	1.043187
-160	1.035071
-159	1.027522
-158	1.022928
-157	1.017598
-156	1.011779
-155	1.005592
-154	0.999145
-153	0.992163
-152	0.985240
-151	0.976933
-150	0.972630
-149	0.967985
-148	0.962415
-147	0.957304
-146	0.950985
-145	0.944818
-144	0.938498
-143	0.932083
-142	0.924901
-141	0.918310
-140	0.911451
-139	0.904035
-138	0.896807
-137	0.889958
-136	0.882549
-135	0.875279
-134	0.867705

```
-133 0.860456
-132 0.852854
-131 0.845260
-130 0.837612
-129 0.829686
-128 0.821769
-127 0.813722
-126 0.809685
-125 0.804592
-124 0.798698
-123 0.792605
-122 0.786704
-121 0.780308
-120 0.773511
-119 0.766965
-118 0.760475
-117 0.753243
-116 0.745048
-115 0.740137
-114 0.735760
-113 0.730755
-112 0.724890
-111 0.719085
-110 0.713179
-109 0.706749
-108 0.700470
-107 0.694408
-106 0.687506
-105 0.680996
-104 0.674370
-103 0.667865
-102 0.661165
-101 0.653680
-100 0.646807
-99 0.639454
-98 0.632456
-97 0.623816
-96 0.620223
-95 0.615391
-94 0.610500
-93 0.604716
-92 0.598960
-91 0.592436
-90 0.586447
-89 0.580037
-88 0.573215
-87 0.566507
-86 0.559349
-85 0.552256
-84 0.545450
-83 0.538047
-82 0.531168
-81 0.523893
-80 0.516170
-79 0.508904
-78 0.501207
```

-77	0.493472
-76	0.486089
-75	0.478353
-74	0.470615
-73	0.462502
-72	0.454250
-71	0.445037
-70	0.439015
-69	0.437337
-68	0.434006
-67	0.429830
-66	0.424853
-65	0.419425
-64	0.413964
-63	0.407755
-62	0.401676
-61	0.395172
-60	0.388079
-59	0.381077
-58	0.373804
-57	0.366523
-56	0.358970
-55	0.351027
-54	0.343273
-53	0.335210
-52	0.326852
-51	0.318647
-50	0.310299
-49	0.301366
-48	0.294665
-47	0.289034
-46	0.282158
-45	0.277616
-44	0.273728
-43	0.268590
-42	0.262926
-41	0.256752
-40	0.250021
-39	0.243020
-38	0.236023
-37	0.228442
-36	0.221041
-35	0.213375
-34	0.205688
-33	0.198079
-32	0.190291
-31	0.181997
-30	0.174913
-29	0.173851
-28	0.170501
-27	0.166203
-26	0.161157
-25	0.155605
-24	0.149582
-23	0.143249
-22	0.136497

-21	0.129725
-20	0.122647
-19	0.115441
-18	0.108077
-17	0.100446
-16	0.094786
-15	0.089923
-14	0.083954
-13	0.078748
-12	0.074825
-11	0.069535
-10	0.063419
-9	0.056557
-8	0.049103
-7	0.041166
-6	0.033846
-5	0.028592
-4	0.024332
-3	0.019160
-2	0.012224
-1	0.007283
